# Electroforming-Free Bipolar Resistive Switching Memory Based on Magnesium Fluoride

**DOI:** 10.3390/mi12091049

**Published:** 2021-08-30

**Authors:** Nayan C. Das, Minjae Kim, Jarnardhanan R. Rani, Sung-Min Hong, Jae-Hyung Jang

**Affiliations:** 1 School of Electrical Engineering and Computer Science, Gwangju Institute of Science and Technology, Gwangju 61005, Korea; nayan@gist.ac.kr (N.C.D.); min7kim9@gist.ac.kr (M.K.); rani@gist.ac.kr (J.R.R.); smhong@gist.ac.kr (S.-M.H.); 2 School of Energy Technology, Korea Institute of Energy Technology, Naju 58330, Korea

**Keywords:** electroforming-free, bipolar, RRAM, filamentary switching, interface

## Abstract

Electroforming-free resistive switching random access memory (RRAM) devices employing magnesium fluoride (MgF_x_) as the resistive switching layer are reported. The electroforming-free MgF_x_ based RRAM devices exhibit bipolar SET/RESET operational characteristics with an on/off ratio higher than 10^2^ and good data retention of >10^4^ s. The resistive switching mechanism in the Ti/MgF_x_/Pt devices combines two processes as well as trap-controlled space charge limited conduction (SCLC), which is governed by pre-existing defects of fluoride vacancies in the bulk MgF_x_ layer. In addition, filamentary switching mode at the interface between the MgF_x_ and Ti layers is assisted by O–H group-related defects on the surface of the active layer.

## 1. Introduction

Among emerging non-volatile memory (NVM) technologies, resistive switching random access memory (RRAM) is a promising technology due to its properties of low-power consumption, high switching speed, excellent scalability, long-endurance, simple architecture, and complementary metal-oxide-semiconductor (CMOS) technology compatibility [1]. RRAM can also mimic biological synopsis, showing potential for neuromorphic applications [2].

RRAMs are based on a metal-insulator-metal (MIM) structure, which generally involves an electroforming process to create conductive filaments between two metal electrodes through the insulating layer. After the electroforming process, the device switches from an initial high resistance state (HRS) to a low resistance state (LRS). Other device operation parameters, including the applied voltage/current and the device operating environment, strongly affect the electroforming process [3]. Usually, the voltage required for formation is much higher than that required for the switching operation, which causes unexpected complexity and worsens device performance [4]. Hence, electroforming-free resistive switching is one of the most desired characteristics of RRAM.

Electroforming-free behavior is usually considered to result from internal defects and conduction filament confinement [5]. It is often observed in devices consisting of nonstoichiometric metal oxides due to oxygen ion migration and the presence of defects [6,7]. Defect profile manipulation, ion doping [5], active layer thickness optimization [8], thermal treatment [9], and fabrication process modulation have been studied [5,10] to achieve electroforming-free characteristics.

Biodegradable materials (biopolymers and biomass) based on RRAM devices have demonstrated promising results. The advantage of biodegradable materials is non-toxic and eco-friendly, significantly reducing the difficulty of handling electronic waste and alleviating the related problems [11]. However, the essential material properties still remain significant issues in biomaterial-based RRAMs. A thick active layer is required for the better performance in terms of reliability and uniformity. Magnesium fluoride (MgF_x_), having a rutile crystal structure, is a biodegradable metal fluoride and an insulator with a large bandgap (11.3 eV). Its lower degradation rate compared to other biodegradable metals and polymers make it a favorable candidate for biodegradable RRAM devices [12,13].

However, the devices require an electroforming process to activate the resistive switching properties. Electroforming voltages as high as 20 V are required [13]. The electroforming-free MgF_x_ based RRAM device has not yet been reported. In addition, there is limited information about the characteristics of the MgF_x_ active layers. The conduction mechanism and resistive switching mechanism in MgF_x_ based RRAM devices are not fully understood either.

In this work, electroforming-free Ti/MgF_x_/Pt RRAM devices are synthesized and characterized. The devices exhibit bipolar resistive switching behavior with an on/off ratio higher than 10^2^. Structural, elemental, and compositional characteristics of the MgF_x_ thin film are thoroughly investigated. The electroforming-free behavior is introduced in the device by deliberately making the amorphous MgF_x_ layer contain more defects than the crystalline layer. The amorphous MgF_x_ layer is deposited by the electron beam (e-beam) evaporation method at room temperature.

The effects of device size and MgF_x_ thickness on the overall device performance are systematically investigated to explore the conduction and resistive switching mechanisms. Finally, with a feasible conduction and resistive switching model, mechanisms are presented in detail.

## 2. Materials and Methods

Ti/MgF_x_/Pt devices were fabricated on SiO_2_/Si substrate. A 150-nm-thick Pt bottom electrode was deposited by e-beam evaporation. A thin layer of Ti was used as an adhesion layer between Pt and the SiO_2_/Si substrate. A circular-shaped shadow mask was utilized to pattern the variable thickness MgF_x_ and 150-nm-thick Ti top electrode during e-beam evaporation at room temperature.

Then, 50-nm-thick MgF_x_ based devices having four different top electrode radii of 25, 50, 150, and 225 μm were prepared to observe the area dependency. The 50 μm-radius devices having 30, 50, and 70-nm-thick MgF_x_ layers were fabricated to investigate the effect of the MgF_x_ thickness. Separately, 50-nm and 1-μm-thick MgF_x_ films were grown on glass and silicon substrates for X-ray diffraction (XRD; panalytical x’pert pro, Almelo, Netherlands) analysis, scanning electron microscope (SEM; Hitachi S-4700, Hitachi High-Tech Corporation, Tokyo, Japan) analysis, X-ray photoelectron spectroscopy (XPS; NEXSA, Fisher Scientific, 168 Third Avenue. Waltham, MA, USA), and Fourier transform infrared (FTIR; Bruker/Vertex 80v, Bruker Optics Inc, Billerica, MA, USA) absorbance spectroscopy measurement. The electrical characteristics of the memory devices were measured using a semiconductor parameter analyzer (HP-4155A; Hewlett-Packard Company, 3000 Hanover Street, Palo Alto, CA, USA). Voltage was applied directly to the top electrode while the bottom electrode was grounded.

## 3. Results and Discussion

### 3.1. Characteriazation of MgF_x_ Thin Film

**Figure 1:**
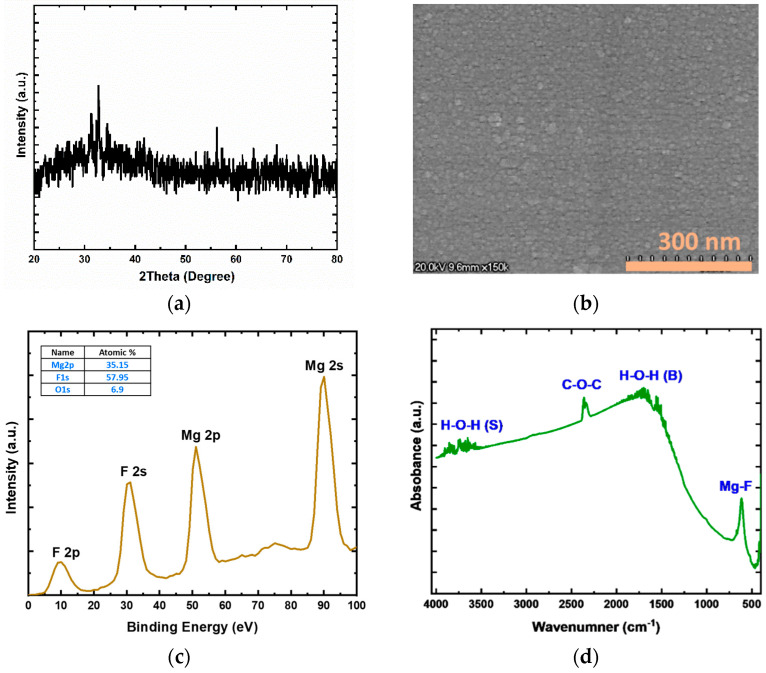
Structural and compositional analysis of MgF_x_ thin film (**a**) XRD pattern of MgFx film; (**b**) SEM image of the surface; (**c**) XPS analysis with characteristics peaks and atomic percentages of magnesium and fluorine; (**d**) FTIR absorbance spectra.

The structural, elemental, and compositional properties of the MgF_x_ thin film are studied to understand the overall Ti/MgF_x_/Pt device performance, resistive switching, and conduction mechanism. Figure 1 shows XRD pattern, SEM image, XPS analysis, and FTIR absorbance spectroscopy measurement results for the MgF_x_ thin film.

The structures of MgF_x_ thin films can be controlled in phases from amorphous to crystalline by increasing the substrate temperature during deposition from room temperature to 300 °C [14,15]. The substrate temperature was kept at ambient temperature during e-beam deposition to fabricate a defect-rich amorphous MgF_x_ active layer.

The XRD pattern of the MgF_x_ thin-film proves that this film is amorphous, as shown in Figure 1a. The SEM image of the MgF_x_ thin film in Figure 1b shows that only small grains were formed. The XRD and SEM analyses revealed that the amorphous granular structured MgF_x_ layer was successfully realized.

Figure 1c shows XPS analysis results for the MgF_x_ thin film. The characteristic peak of MgF_x_ is confirmed by the peak position of Mg 2p at 52 eV [12]. Via curve-fitting and area analysis, the atomic ratio of Mg to F is around 1:1.65. The deficiency of F shows the presence of fluoride vacancies in the MgF_x_ layer. A small amount of oxygen is also observed [16].

The FTIR absorbance spectra analysis result for the MgF_x_ thin film is shown in Figure 1d. The characteristic absorbance peak at 613 cm^−1^ is assigned to the Mg–F bond. During fabrication, because it is amorphous, MgF_x_ can absorb H_2_O from the atmosphere on its surface [15,16,17]. Due to the presence of H_2_O, there are many weak absorption peaks between 3800–3500 cm^−1^ and 1700 and 1450 cm^−1^. The vibration bands at 3800 to 3500 cm^−1^ are associated with the O-H stretching vibrations of water molecules and the weak binding of hydroxyl groups at Mg^2+^ sites. The vibration bands at 1700–1450 cm^−1^ are associated with the bending mode of O–H groups in H_2_O [17,18,19]. Additionally, a weak CO_2_ (gas-phase) vibration band is observed at around 2375–2385 cm^−1^ [19]. Due to higher defects at their grain boundaries, amorphous films generally absorb more moisture than crystalline films [16,17,18,19].

### 3.2. Electrical Characteristics of Ti/MgF_x_/Pt Device

**Figure 2:**
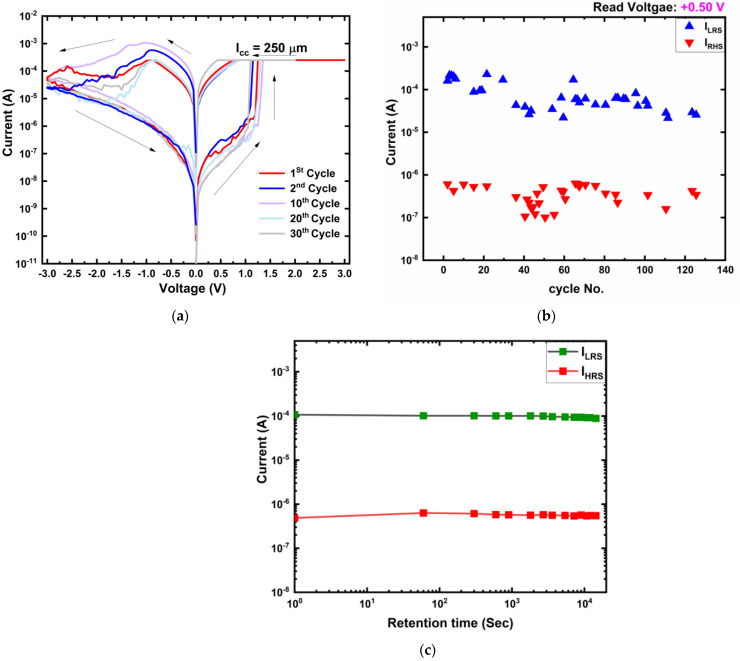
(**a**) Typical I-V characteristics of Ti/MgF_x_/Pt for 1st, 2nd, 10th, 20th, and 30th cycles; (**b**) On and off currents up to 125 cycles. (**c**) Data retention characteristics of the device.

Current-voltage (I-V) measurement of the Ti/MgF_x_/Pt devices was carried out by applying double sweep DC voltage with a 50-mV step. Figure 2a shows five cycles of I-V characteristics (1st, 2nd, 10th, 20th, and 30th) of a 50-nm-thick MgF_x_ based memory device with a 25-μm radius. Figure 2b shows the device is on and off current stability up to 125 cycles. The data retention time of the device is over 10^4^ s as shown in Figure 2c. The first cycle sequence was 0 V → +3 V → 0 V → −3 V → 0 V, which is shown in red. From the second cycle (shown in blue) the sequence was 0 V → +2 V → 0 V → −3 V → 0 V. When the positive voltage was applied to the top electrode, the compliance current (I_cc_) was set to 250 μA to protect the device from permanent breakdown. The I-V curves show hysteretic bipolar resistive switching characteristics.

Pristine Ti/MgF_x_/Pt device is in the high resistance state (HRS), exhibiting resistance higher than 10 MΩ. When positive voltage increases, the device current increases gradually but suddenly increases up to the I_cc_ limit. The resistance switching from HRS to low resistance state (LRS) occurs at around +1.25 V (V_SET_). When a negative voltage is applied, the current starts decreasing from approximately −0.9 V, the RESET voltage (V_RESET_). Current decreases gradually until the negative bias voltage reaches −3 V. The currents in the LRS and HRS of the device are denoted the on current (I_LRS_) and off current (I_HRS_), respectively. A readout voltage (V_Read_) of +0.50 V is utilized to read the I_LRS_ and I_HRS_ values of the device. The I_LRS_/I_HRS_ ratio of the device is higher than 10^2^ even after 125 cycles, as shown in Figure 2b. The device also performs good data retention over 10^4^ s with an on/off ratio higher than 10^2^, as shown in Figure 2c.

In Figure 2a, magnitudes of V_SET_ and V_RESET_ are almost identical for all cycles, including the first cycle, even though the defects in the new devices and after RESET (HRS) are different. In addition, the resistances in HRS for all cycles are close to the initial value (~10 MΩ). These are the two typical characteristics of electroforming-free bipolar resistive switching devices [4,5]. The electroforming-free resistive switching behavior of Ti/MgF_x_/Pt can be attributed to the combination of ample initial fluoride-related defects in the amorphous MgF_x_ layer and the presence of O–H group-related defects at the interface of the Ti/MgF_x_. The O–H group-related defects play significant roles in electroforming-free resistive switching by providing additional charges and facilities for forming anion vacancies in the top electrode/active layer interface [16,20,21,22]. This makes the devices overall less resistive than the other reported devices (~10 GΩ). Moreover, it implies that the conventional electroforming process is not necessary for these MgF_x_ based devices.

### 3.3. Conduction and Resistive Switching Mechanism

The area dependency and thickness dependency of the Ti/MgF_x_/Pt device performance are essential to analyze to confirm the conduction and resistive switching mechanisms.

#### 3.3.1. Area Dependency

**Figure 3:**
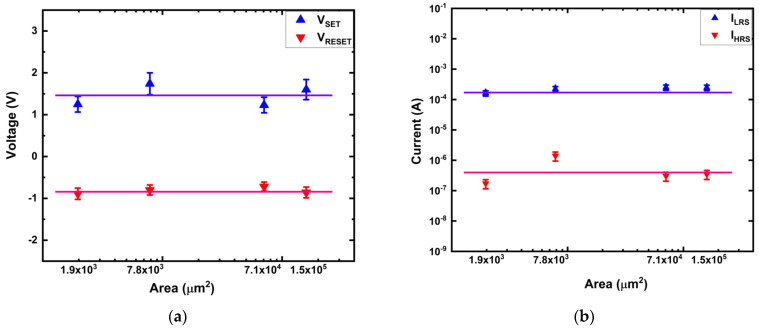
Area dependent characteristics of Ti/MgF_x_/Pt memory devices. (**a**) SET and RESET voltage vs. area; (**b**) On and Off current vs. area.

The area-dependent characteristics of Ti/MgF_x_/Pt memory devices are shown in Figure 3. V_SET_ and V_REST_ are independent of the device area (Figure 3a). Neither I_LRS_ nor I_HRS_ shows any area dependency (Figure 3b), either. These area independent voltages (V_SET_ and V_REST_) and currents (I_LRS_ and I_HRS_) imply that filament type resistive switching takes place in Ti/MgF_x_/Pt devices [23,24].

After the SET process, charge carriers flowed through the conduction filament (CF) instead of the whole device area in the LRS. After the RESET process, current contribution is dominated by thermally activated localized states at the partially ruptured filaments and not by the bulk layer [23,24,25,26,27].

#### 3.3.2. Thickness Dependency

**Figure 4:**
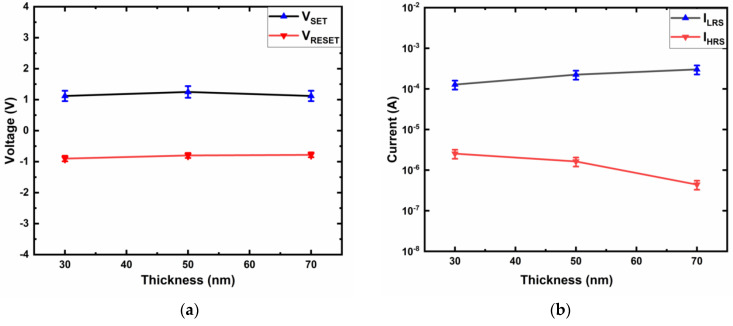
Active layer thickness dependent characteristics of Ti/MgF_x_/Pt memory devices. (**a**) SET and RESET voltage vs. thickness; (**b**) On and Off current vs. thickness.

The active layer thickness-dependent characteristics were investigated to explore the location of CF in the Ti/MgF_x_/Pt memory devices. All the devices with 30, 50, and 70-nm-thick MgF_x_ layers exhibit electroforming-free behavior. Both V_SET_ and V_REST_ are independent of the active layer thickness (Figure 4a). I_LRS_ does not show any thickness dependency, but I_HRS_ decreases as the thickness increases (Figure 4b). These thickness independencies of the voltages (V_SET_ and V_REST_) and currents (I_LRS_) imply the filament type resistive switching of Ti/MgF_x_/Pt devices and show that CF is forming at the interface of Ti/MgF_x_ [28].

The thickness independence of V_SET_ and V_REST_ implies that the voltage drops on the CF mainly takes place at the electrode/dielectric interface [29]. The thickness of the interface region does not considerably change for the bulk thickness. The resistive switching of the device occurs at a local CF type rather than over a whole interfacial area [29,30,31,32,33,34].

The thickness independence of I_LRS_ is attributed to filament-type resistive switching [35]. The I_HRS_ decreases due to the increase in the bandgap with the increase of the active layer thickness causing difficulty of charge carrier movement after the RESET process [36].

Hence, considering the area and thickness independency of Ti/MgF_x_/Pt memory devices, a possible resistive switching mechanism is that the RESET process causes an incomplete rupture of conductive filaments at the Ti/MgF_x_ interface, and the ensuing SET process reconstructs the conductive filaments [29,30,31,32,33,34].

#### 3.3.3. Schematics of Conduction and Switching Mechanism

**Figure 5:**
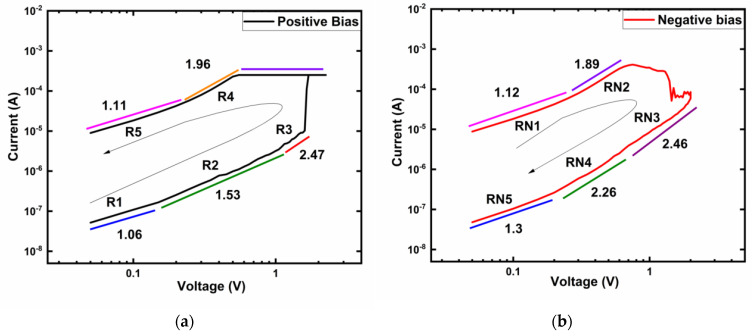
Log (I) − log (V) characteristics of Ti/MgF_x_/Pt memory devices with I_cc_ = 250 μA. (**a**) Positive bias voltage region; (**b**) Negative bias voltage region with slopes of different parts.

A typical I-V curve from Figure 1a was replotted as log(I) − log(V) with curve fittings to determine the conduction mechanism in the resistive switching states of Ti/MgF_x_/Pt devices with the results shown in Figure 5. The positive voltage regions in HRS and LRS are divided into R1, R2, R3, R4, and R5 are shown in Figure 5a. The negative voltage regions in LRS and HRS are divided into RN1, RN2, RN3, RN4, and RN5 to illustrate the conduction process, as shown in Figure 5b.

In the low positive voltage region, the slopes of the fitting lines for both HRS (R1: 1.06) and LRS (R5: 1.11) are close to 1, which implies that ohmic conduction (I ∝ V) dominates in these regions. As the voltage increases, the slopes of both HRS (R3:2.47) and LRS (R4: 1.96) increase. At higher voltages, the conduction mechanism follows Child’s square law (I ∝ V^2^). Additionally, there is a sharp increase of current at the voltage of around +1.25 V. The I-V characteristics in the negative voltage region also show a similar pattern (Figure 5b). These conduction characteristics of LRS and HRS indicate the trap-controlled space charge limited conduction (SCLC) mechanism [37,38,39,40].

**Figure 6:**
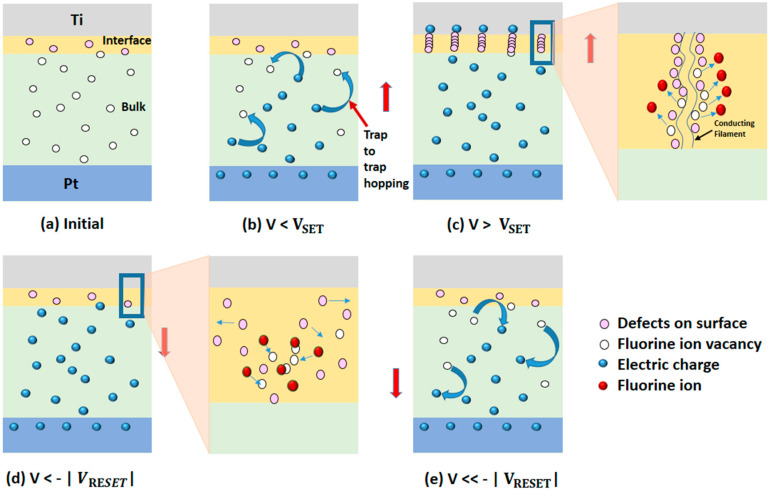
Schematics of proposed switching mechanism of Ti/MgF_x_/Pt memory device. (**a**) Initial, (**b**) V < V_SET_, (**c**) V > V_SET_, (**d**) $V<-\left|V_{RESET}\right|$, (**e**) $V\ll -\left|V_{RESET}\right|$.

Based on the above analysis, the complete resistive switching mechanism of the Ti/MgF_x_/Pt memory device is proposed below, and the step-by-step schematics are shown in Figure 6.

Pristine Ti/MgF_x_/Pt devices contain a considerable number of intrinsic defects in the form of $F^{-1}$ vacancies in bulk MgF_x_ active layer. A small number of extrinsic defects is also present on the surface of the active layer in the form of O–H groups. As a result, the interface region has a different chemistry than the bulk MgF_x_ for defect formation and ultimately produces stochastic defects [21]. All defects (intrinsic and extrinsic) are regarded as traps in Ti/MgF_x_/Pt devices. (Figure 6a).

When a positive voltage is applied, the traps control the SCLC. In the lower voltage region (R1), the injection of electrons is very low. As a result, the conduction is largely dominated by free carriers thermally generated inside the MgF_x_ film.

As the bias voltage goes up, the injection of electrons becomes high. The injected electron concentration gradually surpasses the equilibrium electron concentration in the film and dominates the current conduction in the device. When the voltage reaches near V_SET_, the injected electrons are partly captured by the traps in the MgF_x_ film. Thus, the charge-trapping process creates the conduction paths through the traps in the bulk of the MgF_x_ layer from the bottom electrode to the interface (Figure 6b)

The intrinsic ($F^{-1}$ vacancies) and extrinsic (O–H groups) defects are confined rather than uniformly distributed at the interface. These defects are mostly produced at grain boundaries in the amorphous MgF_x_ active layer. Grain boundaries act as preferred conduction pathways for vacancies, accumulating more effortlessly at the electrode and altering the potential barrier at the electrode/oxide interfaces [12,22,41]. During the SET process, fluoride ions move from the grain boundaries to the top electrode (Ti) due to the applied voltage. The forming and migrating energies of fluoride vacancies in MgF_x_ are approximately 1.44 eV and 0.85 eV [42], which are lower than those of other RRAM devices based on oxygen vacancies [43].

When a positive bias voltage is applied, the O–H groups disassociate into differently charged species (O^2−^and H^+^). This process causes an increase in the conductivity of the interface region [20,22].

At V_SET_, localized CF is formed with the combined contribution of fluoride vacancies, oxygen vacancies, and protons in the interfacial region between the Ti and MgF_x_ layers [28]. As a result, the device resistance state switches from HRS to LRS (Figure 6c).

In the LRS state, O–H group-related defects improve electron hopping by providing additional hopping paths in the surrounding Ti/ MgF_x_ interface. However, far from the interface, the hopping mechanism remains unaffected in most of the bulk MgF_x_. Therefore, I_LRS_ barely increases and remains constant with thickness variation [21]. As the positive voltage sweeps back, most of the injected electrons are free carriers due to the occupied traps and the device maintains LRS.

When a negative voltage is applied at V_RESET_, fluoride ions and oxygen ions migrate back to the CF and gradually recombine with the vacancies. As a result, the CF in the interface is ruptured [44,45]. O^2−^ and H^+^ form O–H groups and attach to Mg^2+^ sites. The absorption of O–H groups in Mg^2+^ sites is a reversible process [17,18,19,20]. Thus, O–H group-related traps also decrease during negative bias. Consequently, the MgF_x_ active layer becomes resistive, and the overall device resistance state gradually changes from LRS to HRS (Figure 6d).

As the negative voltage increases further, de-trapping of the electrons becomes noticeable. Conduction paths in bulk MgF_x_ are damaged by the charge de-trapping process. The trapped electrons bounce back through the fluoride-related traps of the MgF_x_ layer from the interface to the bottom [4,13]. As a result, the resistance state of the device switches to HRS, following the trap-controlled SCLC mechanism (Figure 6e).

## 4. Conclusions

A magnesium fluoride (MgF_x_)-based nonvolatile resistive switching memory device demonstrating electroforming-free, bipolar switching characteristics was successfully fabricated by employing Ti and Pt as top and bottom electrodes, respectively. The device exhibits a stable resistive switching property with data retention of >10^4^ s with an on/off ratio > 10^2^. The resistive switching mechanism is explored by thorough structural and compositional analyses of MgF_x_ thin films and performance analysis of the device with size and MgF_x_ thickness variations.

A sufficient amount of fluoride vacancies in bulk and the existence of O–H group-related defects at the interface are the sources of the electroforming-free characteristics of the device. These intrinsic and extrinsic defects are the sources of the resistive switching mechanism, operating by charge trapping and de-trapping in the bulk amorphous MgF_x_ layer and by the formation and rupture of CF at the Ti/MgF_x_ interface region.

## References

[1] Prakash A., Jana D., Maikap S. (2013). TaOx-based resistive switching memories: Prospective and challenges. Nanoscale Res. Lett..

[2] Upadhyay N.K., Jiang H., Wang Z., Asapu S., Xia Q., Joshua Yang J. (2019). Emerging Memory Devices for Neuromorphic Computing. Adv. Mater. Technol..

[3] Jeong D.S., Schroeder H., Breuer U., Waser R. (2008). Characteristic electroforming behavior in Pt/TiO_2_/Pt resistive switching cells depending on atmosphere. J. Appl. Phys..

[4] Das N.C., Oh S.I., Rani J.R., Hong S.M., Jang J.H. (2020). Multilevel bipolar electroforming-free resistive switching memory based on silicon oxynitride. Appl. Sci..

[5] Fang Z., Yu H.Y., Li X., Singh N., Lo G.Q., Kwong D.L. (2011). HfO_x_/TiO_x_/HfO_x_/TiO_x_ multilayer-based forming-free RRAM devices with excellent uniformity. IEEE Electron Device Lett..

[6] Wan Z., Darling R.B., Majumdar A., Anantram M.P. (2017). A forming-free bipolar resistive switching behavior based on ITO/V_2_O_5_/ITO structure. Appl. Phys. Lett..

[7] Wong H.S.P., Lee H.Y., Yu S., Chen Y.S., Wu Y., Chen P.S., Lee B., Chen F.T., Tsai M.J. (2012). Metal-oxide RRAM. Proc. IEEE.

[8] Chen Y.S., Lee H.Y., Chen P.S., Wu T.Y., Wang C.C., Tzeng P.J., Chen F., Tsai M.J., Lien C. (2010). An ultrathin forming-free HfO_x_ resistance memory with excellent electrical performance. IEEE Electron Device Lett..

[9] Kawai M., Ito K., Ichikawa N., Shimakawa Y. (2010). Thermally formed conducting filaments in a single-crystalline NiO thin film. Appl. Phys. Lett.

[10] Cao X., Li X., Gao X., Yu W., Liu X., Zhang Y., Chen L., Cheng X. (2009). Forming-free colossal resistive switching effect in rare-earth-oxide films for memristor applications. J. Appl. Phys.

[11] Park S.P., Kim H.J., Lee J.H., Kim H.J. (2019). Glucose-based resistive random access memory for transient electronics. J. Inf. Disp..

[12] Zhang Z., Tsang M., Chen I.W. (2016). Biodegradable resistive switching memory based on magnesium difluoride. Nanoscale.

[13] Sun Y., Wang C., Xu H., Song B., Li N., Li Q., Liu S. (2019). Transition from rectification to resistive-switching in Ti/MgF_2_/Pt memory. AIP Adv..

[14] Yang H.-H., Park G.-C. (2010). A Study on the Properties of MgF_2_ Antireflection Film for Solar Cells. Trans. Electr. Electron. Mater..

[15] Pilvi T., Hatanpää T., Puukilainen E., Arstila K., Bischoff M., Kaiser U., Kaiser N., Leskelä M., Ritala M. (2007). Study of a novel ALD process for depositing MgF_2_ thin films. J. Mater. Chem..

[16] Valov I., Tsuruoka T. (2018). Effects of moisture and redox reactions in VCM and ECM resistive switching memories. J. Phys. D Appl. Phys..

[17] Dumas L., Quesnel E., Robic J.-Y., Pauleau Y. (2000). Characterization of magnesium fluoride thin films deposited by direct electron beam evaporation. J. Vac. Sci. Technol. A Vac. Surf. Films.

[18] Wuttke S., Vimont A., Lavalley J.C., Daturi M., Kemnitz E. (2010). Infrared investigation of the acid and basic properties of a sol-gel prepared MgF_2_. J. Phys. Chem. C.

[19] Selvam N.C.S., Kumar R.T., Kennedy L.J., Vijaya J.J. (2011). Comparative study of microwave and conventional methods for the preparation and optical properties of novel MgO-micro and nano-structures. J. Alloys Compd..

[20] Lübben M., Wiefels S., Waser R., Valov I. (2018). Processes and Effects of Oxygen and Moisture in Resistively Switching TaO_x_ and HfO_x_. Adv. Electron. Mater..

[21] Gao R., Lei D., He Z., Chen Y., Huang Y., En Y., Xu X., Zhang F. (2020). Effect of Moisture Stress on the Resistance of HfO_2_/TaO_x_-Based 8-Layer 3D Vertical Resistive Random Access Memory. IEEE Electron Device Lett..

[22] Bagdzevicius S., Maas K., Boudard M., Burriel M. (2017). Interface-type resistive switching in perovskite materials. J. Electroceram..

[23] Kurinec S.K., Iniewski K. (2013). Nanoscale Semiconductor Memories: Technology and Applications.

[24] Ielmini D., Spiga S., Nardi F., Cagli C., Lamperti A., Cianci E., Fanciulli M. (2011). Scaling analysis of submicrometer nickel-oxide-based resistive switching memory devices. J. Appl. Phys..

[25] Lu Y., Lee J.H., Yang X., Chen I.W. (2016). Distinguishing uniform switching from filamentary switching in resistance memory using a fracture test. Nanoscale.

[26] Song Y.L., Liu Y., Wang Y.L., Wang M., Tian X.P., Yang L.M., Lin Y.Y. (2011). Low reset current in stacked AlO_x_/WO_x_ resistive switching memory. IEEE Electron Device Lett..

[27] Lu Y., Lee J.H., Chen I.W. (2017). Scalability of voltage-controlled filamentary and nanometallic resistance memory devices. Nanoscale.

[28] Traore B., Blaise P., Sklenard B., Vianello E., Magyari-Kope B., Nishi Y. (2018). HfO_2_/Ti Interface Mediated Conductive Filament Formation in RRAM: An Ab Initio Study. IEEE Trans. Electron Devices.

[29] Janousch M., Meijer G.I., Staub U., Delley B., Karg S.E., Andreasson B.P. (2007). Role of oxygen vacancies in cr-doped SrTiO_3_ for resistance-change memory. Adv. Mater..

[30] Schindler C., Staikov G., Waser R. (2009). Electrode kinetics of Cu-SiO_2_-based resistive switching cells: Overcoming the voltage-time dilemma of electrochemical metallization memories. Appl. Phys. Lett..

[31] Inoue I.H., Yasuda S., Akinaga H., Takagi H. (2008). Nonpolar resistance switching of metal/binary-transition-metal oxides/metal sandwiches: Homogeneous/inhomogeneous transition of current distribution. Phys. Rev. B Condens. Matter Mater. Phys..

[32] Zhang Y.P., Wang H., Xu J.W., Yang L., Qiu W., Li Z. (2014). Da Effect of ZnMn_2_O_4_ thickness on its resistive switching characteristics. Indian J. Eng. Mater. Sci..

[33] Yang Y.C., Pan F., Zeng F. (2010). Bipolar resistance switching in high-performance Cu/ZnO: MMn/Pt nonvolatile memories: Active region and influence of Joule heating. New J. Phys..

[34] Yang J.J., Pickett M.D., Li X., Ohlberg D.A.A., Stewart D.R., Williams R.S. (2008). Memristive switching mechanism for metal/oxide/metal nanodevices. Nat. Nanotechnol..

[35] Chen Y.J., Chen H.L., Young T.F., Chang T.C., Tsai T.M., Chang K.C., Zhang R., Chen K.H., Lou J.C., Chu T.J. (2014). Hydrogen induced redox mechanism in amorphous carbon resistive random access memory. Nanoscale Res. Lett..

[36] Wang L.P., De Han P., Zhang Z.X., Zhang C.L., Xu B.S. (2013). Effects of thickness on the structural, electronic, and optical properties of MgF_2_ thin films: The first-principles study. Comput. Mater. Sci..

[37] Chiu F.C. (2014). A review on conduction mechanisms in dielectric films. Adv. Mater. Sci. Eng..

[38] Li Y.T., Long S.B., Liu Q., Lü H.B., Liu S., Liu M. (2011). An overview of resistive random access memory devices. Chin. Sci. Bull..

[39] Lim E.W., Ismail R. (2015). Conduction mechanism of valence change resistive switching memory: A survey. Electronics.

[40] Chiu F.C., Chou H.W., Lee J.Y.M. (2005). Electrical conduction mechanisms of metal La_2_O_3_ Si structure. J. Appl. Phys..

[41] Simanjuntak F.M., Ohno T., Samukawa S. (2019). Film-Nanostructure-Controlled Inerasable-to-Erasable Switching Transition in ZnO-Based Transparent Memristor Devices: Sputtering-Pressure Dependency. ACS Appl. Electron. Mater..

[42] Park D.S., Nowick A.S. (1976). Ionic conductivity and point defects in pure and doped MnF_2_ and MgF_2_ single crystals. J. Phys. Chem. Solids.

[43] Field B.R., Ielmini D. (2011). Modeling the Universal Set/Reset Characteristics of Filament Growth. IEEE Trans. Electron Devices.

[44] Yuan X.C., Tang J.L., Zeng H.Z., Wei X.H. (2014). Abnormal coexistence of unipolar, bipolar, and threshold resistive switching in an Al/NiO/ITO structure. Nanoscale Res. Lett..

[45] Lin C.Y., Wu C.Y., Wu C.Y., Tseng T.Y., Hu C. (2007). Modified resistive switching behavior of ZrO_2_ memory films based on the interface layer formed by using Ti top electrode. J. Appl. Phys..

